# Do Human Fetuses Form Long‐Lasting Chemosensory Memories? Longitudinal Follow‐Up From Fetus to Young Child of Facial Responses to Flavor/Odor Stimuli

**DOI:** 10.1002/dev.70165

**Published:** 2026-05-12

**Authors:** Nadja Reissland, Jochen Einbeck, Deimantė Baguckaitė, Beyza Ustun‐Elayan, Benoist Schaal, Jacqueline Blissett

**Affiliations:** ^1^ Department of Psychology Durham University Durham UK; ^2^ Department of Mathematical Sciences Durham University Durham UK; ^3^ Department of Psychology University of Cambridge, Old Cavendish Laboratory Cambridge UK; ^4^ Department of Medical Neuroscience Radboud University Medical Center, Donders Institute for Brain, Cognition and Behaviour Nijmegen The Netherlands; ^5^ DOCC Lab, Centre for Taste, Smell and Feeding Behaviour Science Dijon France; ^6^ Université Bourgogne Europe, CNRS, Institut Agro Dijon, Inrae Dijon France; ^7^ School of Psychology, Institute of Health and Neurodevelopment, College of Health and Life Sciences Aston University Birmingham UK

## Abstract

Prenatal flavor exposure is known to provide the foundation for later flavor or odor preferences in humans, yet the persistence of fetal flavor/odor memories into early childhood remains unclear. In previous studies, fetuses at 32‐ and 36 weeks gestation were shown to display discriminative facial reactions following a single‐dose flavor stimulation (bitter kale vs. non‐bitter carrot), and repeated exposure to these flavors from 36 weeks until birth was associated with responses to flavor‐specific odors in the first postnatal month. The current longitudinal follow‐up tested whether the prenatal exposure to two specific flavors results in flavor‐specific odor reactions at 3 years of age. Children (*n =* 12) who had participated in the fetal and neonatal phases of the study were tested at 3 years of age using controlled olfactory presentations of the same flavors. Results showed that the 3‐year‐olds exhibited a significantly reduced rate of negative facial expressions in response to the odor they had been repeatedly exposed to in utero (*p* < 0.001), indicating continuity from prenatal sensory experience to early childhood behavioral responses. Thus, flavor exposure in late gestation can result in long‐lasting flavor/odor memory, confirming that the prenatal chemosensory environment can shape behavioral tendencies years after birth.

## 1 Introduction

The perception of flavors begins in utero with gustatory and olfactory systems developing in the second trimester. By late gestation, chemosensory systems are functional, enabling the fetus to experience flavors from the maternal environment including her diet via the amniotic fluid. This prenatal exposure may form postnatal flavor/odor sensitivities, memories, and preferences and shape related learning and later dietary preferences. Flavor experience, which is an integration of gustation, olfaction, and chemesthesis (trigeminal chemoreception), starts in utero. Fetal taste buds are fully developed by the third trimester of pregnancy, enabling the detection of the taste component of flavors (e.g., Ventura and Worobey 2013). Fetuses also possess a functioning olfactory system, which is connected to higher structures in the brain by 32–35 weeks of gestation (Forestell 2024; Schaal et al. 1995).

Flavors transferred from the mother's diet into the amniotic fluid during pregnancy can thus be engaged in the commencing process of flavor learning. Multiple flavors have been assessed for their ability to pass through the placenta and collect in the amniotic fluid, including garlic, anise, menthol, ethanol, liquorice, carrot, kale, and vanilla (Anunziata et al. 2020; Faas et al. 2000; Gellrich et al. 2024; Mennella et al. 1995; Schaal et al. 2000; Schaal 2023; Spahn et al. 2019). For example, Hepper (1995) claimed that 20‐h‐old neonates whose mothers had consumed garlic during the last month of their pregnancy displayed less aversive oro‐facial responses to garlic odor. Furthermore, a group of neonates exposed to anise in the final 2 weeks of pregnancy showed positive reactions to anise odor when they were between 3 h and 4 days of age, compared with non‐exposed neonates (Schaal et al. 2000). At 6 months of age infants, whose mothers consumed carrot juice during the last month of gestation reacted more positively to carrot‐flavored cereals, compared with plain cereals (Mennella et al. 2001). Similarly, at 8 months of age, infants whose mothers reported consuming quantities of green vegetables, cheese, or fish during pregnancy showed more mouthing indicative of liking when presented with corresponding representative odorants (Wagner et al. 2019). Long‐term effects have also been observed. Hepper et al. (2013) reported that children aged 8–9 years old whose mothers had consumed garlic during pregnancy preferred garlic‐flavored potato purée, compared to children who had not been exposed to garlic. In sum, these findings indicate that prenatal exposure to given flavors can shape subsequent reactions to flavors in human infants, suggesting that in utero chemosensory experience lays the foundation for long term effects on flavor perception and possibly food preference. These results in the human case are backed by a multiplicity of robust experimental studies on various animal models that all found that fetal flavor experience leaves memory imprints that can influence behavior in later development, ranging into adulthood (Dang et al. 2022; Pautassi et al. 2026; Smotherman and Robinson 1990; Stickrod et al. 1982; Villalba et al. 2018; Youngentob et al. 2007).

While previous research has largely focused on the flavor composition of the maternal diet during pregnancy and its association with postnatal reactions to various flavors, few studies have experimentally followed up the effects of prenatally controlled flavor exposure and fetal responses to these flavors across successive stages from fetal to neonatal to early childhood development. In our earlier work, we demonstrated that fetuses displayed initial discriminative behavioral responses to single‐dose flavor stimuli (bitter kale vs. non‐bitter carrot capsules) in late gestation (Ustun et al. 2022). In a subsequent study, we showed that repeated exposure to these flavors from 36 weeks gestation until birth altered infants’ postnatal reactions during the first month of life: Flavors repeatedly experienced in utero elicited fewer negative responses, consistent with familiarity‐driven short‐term memory (Ustun et al. 2025). The current study builds on this work by examining whether these prenatally induced familiarity effects persist into early childhood. Specifically, we predicted that at age 3, children would display fewer negative and/or more positive facial responses to the flavor they had repeatedly experienced in utero, compared to a flavor not repeatedly exposed to during late pregnancy.

In addition to our primary hypothesis regarding long‐term flavor memory, acquired prenatally, we explored whether maternal mental health during pregnancy might influence the hedonic valuation of flavors familiarized in utero. Since maternal prenatal stress and depression can alter fetal neurodevelopment, affective reactivity, and associative learning processes (Laurent 2002; Hartman et al. 2023; Reissland et al. 2020, 2021; Wang et al. 2022), we examined whether maternal mental health variables concurrent with the in utero flavor familiarization could affect responsiveness to the flavor‐specific odorants at age 3.

In the present study, we tested fetal reactions to either kale or carrot flavor administered prenatally (at 32 and 36 weeks of gestation) and followed some of these participants up postnatally at 3 weeks and 3 years of age. In the postnatal test period, we tested young children with the odor component of the carrot and kale flavors they received repeatedly in utero and measured their reactions in terms of smile/laughter and cry gestalts. The present work is hence the first study examining longitudinally specific participant's reactions to nutritional flavors from the fetal period to the postnatal period into early childhood.

## 2 Methods

### 2.1 Participants

Thirty‐four healthy fetuses (17 female, 17 male) participated in the study initially (see Table 1 for participation over time from 32 weeks gestation to 3 years of age). Each fetus was first given a single, acute stimulation to either kale or carrot flavor during two ultrasound sessions (32 and 36 weeks of gestation), allowing the observation of fetal immediate behavioral reactions to a specific flavor. Following the second scan, mothers continued to consume capsules containing their assigned flavor (either kale or carrot) on multiple occasions (4 days or more per week) until birth, providing repeated prenatal exposure to that flavor. All mothers were instructed to consume the capsules between 10 a.m. and 3 p.m. each day. This paradigm ensured that each fetus experienced a flavor environment enhanced by either kale or carrot flavor consumption of capsules throughout the final weeks of gestation. Of the total sample, 20 fetuses (12 female, 8 male) were assigned to the repeated carrot exposure condition, and 14 fetuses (5 female, 9 male) were assigned to the repeated kale exposure condition. The children were followed up at two postnatal points: when they were approximately 3 weeks old (0y) and again at 3 years old (3y). During these follow‐up sessions, both kale and carrot odors were presented to each child in a counterbalanced order. By the 3‐year follow‐up, the number of contactable and available participants had reduced to 12. For clarity, we refer to the four measurement time points as 32w and 36w (prenatal measurements at 32 and 36 weeks of gestation), 0y (0 year, 3 weeks post birth), and 3y (3 years post birth). At each of these time points, fetal or child behavioral reactions to the flavor stimuli were coded. To summarize, at 32 weeks and 36 weeks gestation, fetuses were presented with *only one* flavor stimulus of either kale or carrot by maternal ingestion of a capsule (according to the exposure by design), whereas at 0y and 3y, *both* kale and carrot odors were presented as test stimuli. Hence, we could only code the facial expression to one flavor prenatally, but postnatally we could code and analyze facial reactions to both odors. The numbers of resulting facial expressions coded are given in Table 1 in italic font. Each facial expression was eventually coded into two gestalts, in a process as described in detail below.

**TABLE 1:** Number of fetuses/infants, *n*, and resulting facial gestalts *(N)* available for coding across time points.

	Fetuses prenatally exposed	Number of fetuses/infants, *n* *Number and type of facial movements in reaction to stimuli available for coding, (N)*			
Timepoint		32w	36w	0y	3y
Male	17 (8 carrot, 9 kale)	16 *(16)*	14 *(14)*	16 *(30)*	6 *(12)*
Female	17 (12 carrot, 5 kale)	16 *(16)*	16 *(16)*	16 *(31)*	6 *(12)*
Total	34 (20 carrot, 14 kale)	32 *(32)*	30 *(32)*	32 *(61)*	12 *(24)*

The data from the 32w, 36w, and 0y measurement points are available from previous studies, which also summarize their demographic table (Ustun et al. 2022, 2025). This present study contributes new data for the 3y measurement point. Demographic information for the infants included in this measurement point (last column of Table 1) is provided in Table 2.

**TABLE 2:** Children followed up at 3 years of age.

	Prenatal exposure	
Infant demographics	Kale flavor (*n =* 6)	Carrot flavor (*n =* 6)
Sex: *n* female/*n* male	2/4	4/2
Age at test: mean (s.d.), in months mean, in years	33.57 (4.44) 2.8	39.18 (4.26) 3.2

*Note:* Demographic information for the infants is included in the 3‐year‐olds measurement point. Two infants (one male, one female) were included in the 3‐year follow‐up sample who did not contribute data at earlier time points. For these participants, fetal scans at 32 and 36 weeks were collected but could not be coded due to technical or recording quality issues. Their mothers nevertheless consumed carrot capsules and thereby their fetuses were repeatedly exposed to carrot flavor from 36 weeks until birth, but no postnatal measurements were obtained at the 3‐week follow‐up. These infants therefore contributed only to the 3y dataset.

### 2.2 Stimuli and Procedure

At the 3‐year measurement point, both kale and carrot powders were presented as odor stimuli via a cotton Q‐tip randomized over participants. These powders were identical to those included in the capsules consumed by mothers during pregnancy and matched the stimuli used in the 3‐week (0y) postnatal follow‐up (Ustun et al. 2025). To prepare the stimulus, a cotton tip was first saturated with still water and dipped into kale or carrot powder (∼1 tablespoon) until the entire cotton surface was coated. Testing sessions took place in the homes of the children in a quiet, distraction‐free environment. Each child was seated next to the experimenter, with a video camera positioned to capture the facial reactions of the experimenter and the children. Mothers were seated quietly in the room while completing the questionnaires. The experimenter presented the two odors in random order by placing the cotton Q‐tip under the child's nose, without contacting the nose and holding it there until they turned their head away to indicate disengagement. Facial reactions were video recorded for offline coding using the FOMS system.

### 2.3 Behavioral Coding and Variables

We used the same coding convention as those of previous studies (see Reissland et al. 2011, for the development of the coding scheme of smile/laughter and cry gestalts; Ustun et al. 2022, for the coding scheme with fetuses exposed to kale and carrot; Ustun et al. 2025, for the coding scheme with neonates). The original study identified 17 fetal facial movements (FMs) based on FACS (Ekman and Friesen 1978) and Baby FACS (Oster 2007). Additionally, we coded combinations of FMs (“gestalts”) using Artnatomy (Flores 2005) by identifying facial configurations which are classified as “cry” and “smile/laughter” (Reissland et al. 2011). Separate analyses were carried out for the cry‐face and smile/laughter‐face gestalts. Five FMs were coded frame by frame, specifically for the cry‐face gestalt: FM1 (inner brow raiser), FM4 (brow lowerer), FM10 (upper‐lip raiser), FM16 (lower‐lip depressor), and FM20 (lip stretch). Two FMs were coded specifically for the smile/laughter‐face gestalt: FM12 (lip‐corner puller) and FM19 (tongue show). For details on cry and smile/laughter gestalts, see Reissland et al. (2016) and Ustun et al. (2022) and Table S1 in the Supporting Information. All coding was performed by trained coders blind to flavor conditions, and inter‐reliability was assessed by independent coders who were blind to flavor condition and hypotheses (Cohens’ kappa mean 0.84 range 0.83–0.84).

For each observation, the respective count values of FM were set in relation to the length of the source video, in units of quarters of an hour, for modeling purposes. For instance, if for a certain observation, the cry‐face gestalt count was equal to 2, with a video length of 9.41 min, the outcome of interest is 2/(9.41/15) = 3.19 movements per 0.25 h.

### 2.4 Measurements Across Time

Including the new data from 3y olds into the previously available datasets from 32w, 36w, and 0y, a longitudinal dataset was constructed where observations are linked across the four time points by a pseudo‐anonymized identifier (infant ID). There are two longitudinal outcome measurements in this dataset, namely, cry gestalt and smile/laughter gestalt values. For example, for carrot‐exposed fetuses at 0y, there are 31 smile/laughter gestalt and 31 cry gestalt measurements. Based on data of each fetus contributed a *single* measurement, corresponding to their assigned flavor stimulation and subsequent exposure from 36 weeks (either kale or carrot, at 32w and 36w) and at 0y and 3y, all participating infants were presented with *both* odors (kale and carrot), allowing two potential measurements per participant and gestalt. At 0y, this would theoretically result in 64 measurements (2 × 32) per gestalt, but due to variability in the quality of video recordings, only 61 measures were coded (31 for carrot, 30 for kale; see Table 1). Note that the unequal sample sizes across time points do not pose a statistical problem for longitudinal analysis, so that the available outcome data can be used, under a *mild missingness‐at‐random assumption*, without further imputation or adjustment (Hox 2010).

### 2.5 Assessment of Maternal Mental State Variables

Mothers completed the Perceived Stress Scale (PSS; Cohen et al. 1983) and the Hospital Anxiety and Depression Scale (HADS; Zigmond and Snaith 1983) while their children were tested. The PSS is widely used to assess how unpredictable, uncontrollable, and overloaded respondents perceive their lives to be during the past month. Each item is rated on a 5‐point Likert scale ranging from 0 (“*never*”) to 4 (“v*ery often*”). Total scores range from 0 to 40, with higher scores indicating greater perceived stress. Positively phrased items are reverse‐scored prior to summation. The PSS has demonstrated strong internal consistency (Cronbach's α typically 0.78–0.91) and good construct validity across diverse populations. The HADS (Zigmond and Snaith 1983) assesses both anxiety and depressive symptoms. The HADS consists of 14 items comprising two 7‐item subscales measuring anxiety (HADS‐A) and depression (HADS‐D). Items are rated on a 4‐point scale (0–3), yielding subscale scores ranging from 0 to 21, with higher scores indicating more severe symptoms. Scores of 0–7 are considered normal, 8–10 indicate borderline symptoms, and 11–21 suggest clinically significant levels. The HADS has also demonstrated strong internal consistency (Cronbach's α typically 0.82–0.90) and good construct validity across diverse populations.

For two of the children, maternal mental health measures (depression and stress) were missing at the 3‐year‐olds measurement point. However, for these mothers, the mental health scores from the 3‐week measurement point were available so that the missing scores could be imputed by predictions from a linear regression relating scores at 3 years to the 3‐week scores. Furthermore, for two fetuses, the maternal stress values at 32 weeks were missing, and for the same two fetuses, the maternal depression values at 36 weeks were missing. These were imputed via linear regressions from the corresponding maternal health scores at 36 (for stress) and 32 weeks (for depression). These minor imputation steps enabled us to use data from all 34 fetuses for analysis.

### 2.6 Statistical Analyses

The model used for the gestalt counts is as follows:

$$\begin{array}{ccc} \log\;\left(\frac{gestalt}{codelength}\right) & & =\beta_{0}+\sum_{j=1}^{3}\beta_{j}T_{j}+\beta_{4}gender+\beta_{5}stress\\ & & +\;\beta_{7}depression+\beta_{8}exposure\\ & & +\;\beta_{9}memory+u \end{array}$$

where *gestalt* is a count variable (either smile/laughter gestalt or cry gestalt) equipped with a negative binomial (Type II) distribution, and *u* is a fetus‐level random effect accounting for within‐fetus correlation across time. A negative binomial rather than a Poisson model has been used due to the presence of overdispersion in the gestalt counts (even after inclusion of the random effect). In line with common practice in generalized linear model framework for count data (Dobson et al. 2001), a log‐link was used to relate the gestalt count to the predictors, and an offset was used to incorporate the denominator (codelength) into the model. The variables available for the regression analysis are summarized in Table 3. Beside the outcome variables, the time variables, fetal gender, and the maternal mental health variables, this also includes two covariates that are of particular interest to us: *presentation* (which flavor/odor was presented to the fetus or infant as a test stimulus) and *memory*, a derived variable indicating whether the presentation matches the repeated pre‐natal flavor exposure (this is aways equal to 1 prenatally but not postnatally).

**TABLE 3:** Variables available for regression analysis. For categorical variables, values in brackets (*n*) give the number of observations falling in each category.

Variable	Description
cry gestalt	Integer‐valued (outcome)
smile/laughter gestalt	Integer‐valued (outcome)
codelength	Continuous (offset)
time (*T*)	Factor‐valued, with four ordinal levels: $\;T_{0}=$32w (reference category) *(32)*, $\;T_{1}=$36w *(30)*, $\;T_{2}=$ 0y *(61)*, $\;T_{3}=$3y *(24)*
gender	Indicator variable, with 0 = female *(17)*, 1 = male *(17)*
stress	Maternal stress (PSS scale)
depression	Maternal depression (HADS scale)
anxiety	Maternal anxiety (HADS scale)
presentation	Flavor presentation (pre‐or postnatal), with 0 = carrot *(77)*, 1 = kale *(70)*
memory	Indicator variable, with 1 if postnatal presentation matches the prenatal exposure *(104)*, 0 otherwise *(43)*

*Note:* All variables except gender are time‐dependent. For gender, the *n* values are out of total number of fetuses *(34)*. For the categorical variables, the *n* values are out of total number of observations *(147)*.

The analyses were carried out separately for smile/laughter gestalt and cry gestalt, and models were fitted using R package glmmTMB (McGillycuddy et al. 2025). Full R code for analysis as well as the source data are provided in the Supporting Information.

## 3 Results

### 3.1 Descriptive Analyses

Descriptive summary statistics for all outcome and mental health variables across time are provided in Table 4.

**TABLE 4:** Descriptive statistics (continuous variables) across time points; mean (SD).

Variable	32w	36w	0y	3y
cry gestalt	1.72 (2.99)	1.67 (2.56)	1.66 (3.04)	0.83 (0.96)
smile/laughter gestalt	8.41 (6.06)	10.87 (10.56)	7.05 (5.46)	0.92 (1.28)
codelength	0.71 (0.35)	0.77 (0.29)	0.95 (30)	0.21 (0.08)
stress*	11.72 (5.84)	12.43 (5.66)	11.84 (6.57)	16.37 (5.25)
depression*	4.09 (2.04)	5.05 (2.73)	3.13 (2.78)	4.67 (2.93)
anxiety*	5.94 (2.24)	6.90 (2.82)	5.06 (3.33)	6.47 (3.69)

*Note:* The respective underlying sample sizes are 32, 30, 61, 24 in the first three rows, and 32, 30, 32, 12 in the rows labeled with a *.

**FIGURE 1:**
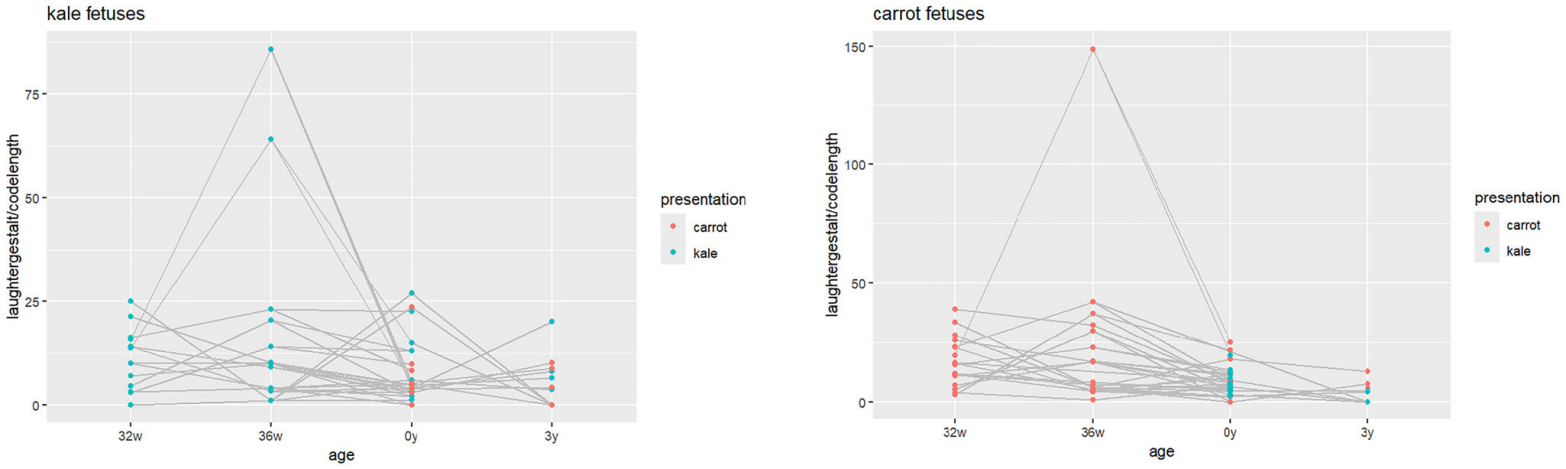
Smile/laughter gestalt relative to coding length for kale‐stimulated fetuses (left) and carrot‐stimulated fetuses (right). Successive measurements relating to the same fetus are connected through gray straight lines. Presentations with kale are symbolized in green and presentations with carrot in red.

**FIGURE 2:**
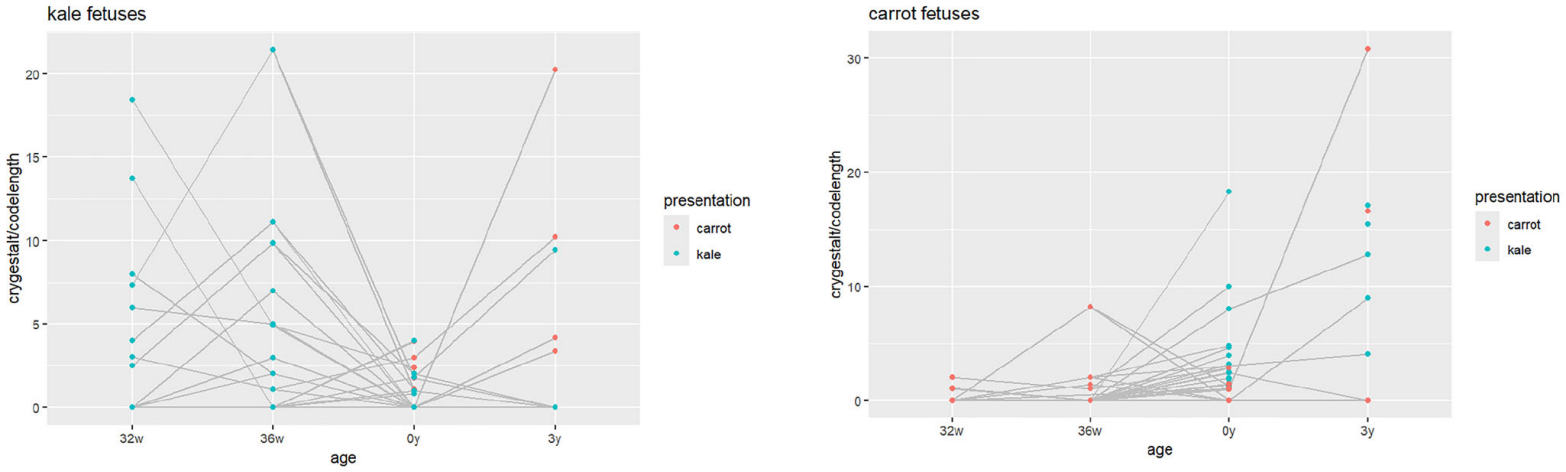
Cry gestalt relative to codelength for kale‐stimulated fetuses (left) and carrot‐stimulated fetuses (right). Successive measurements relating to the same fetus are connected through gray straight lines. Presentations with kale are symbolized in green and presentations with carrot in red.

The smile/laughter gestalt measurements relative to code length are displayed in Figure 1 (left) for fetuses stimulated (at 32w and 36w) to carrot and in Figure 1 (right) for fetuses stimulated with kale. The cry gestalt measurements relative to code length are displayed in Figure 2 (left) for fetuses stimulated with carrot flavor and in Figure 2 (right) for fetuses stimulated with kale flavor. The first two columns in each plot always have the same color, corresponding to the pre‐natal flavor stimulation, either kale or carrot. In the follow‐up at 0y and 3y, both kale and carrot odors are presented to each available infant, hence yielding “two colors” in the plot. For the *smile/laughter gestalt*, the plots indicate that a postnatal flavor presentation resulted in fewer movements when it *differed* from the stimulation flavor. For the *cry gestalt*, a clearer picture emerges that a postnatal presentation differing from the prenatal flavor tended to result in more movements.

**FIGURE 3:**
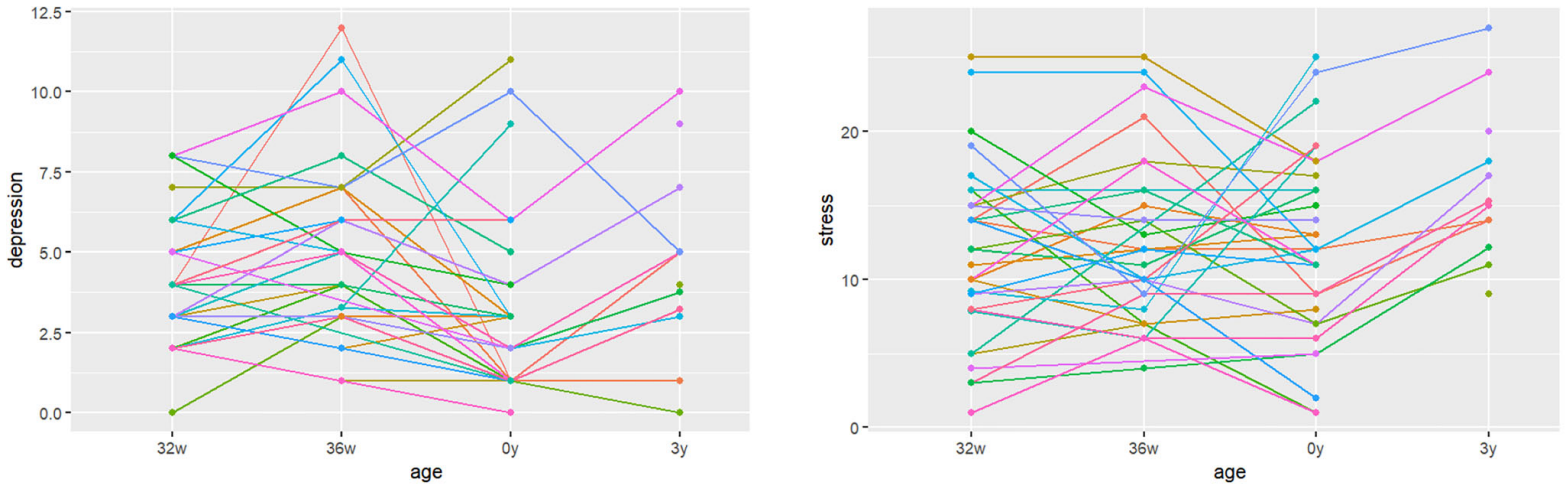
Maternal depression (left) and stress scores (right) across measurement points. A notable tendency for relatively higher depression scores at 36w, and relatively higher stress scores at 3y can be observed from these plots. The corresponding plot for the anxiety variable can be seen in the Supporting Information.

Maternal mental health scores were positively correlated, with a correlation of *r* = 0.60 (*p* < 0.001) for anxiety and depression, *r* = 0.63 (*p* < 0.001) for anxiety and stress, and *r* = 0.53 (*p* < 0.001) for stress and depression. To avoid multicollinearities, only stress and depression were retained as covariates for the model‐based analyses. The longitudinal maternal mental health scores for depression and stress are visualized in Figure 3 (left: depression; right: stress). A notable tendency for relatively higher depression scores at 36w and relatively higher stress scores at 3y can be observed from these plots. The corresponding plot for the anxiety variable can be seen in the Supporting Information.

### 3.2 Modeling Results

Coefficients, standard errors, and *p*‐values from the fitted models for smile/laughter gestalt and cry gestalt movements are provided in Tables 5 and 6, respectively. Full model outputs can be found in the Supporting Information.

**TABLE 5:** Coefficients from the model for the smile/laughter gestalt.

	Coefficient	SE	*p‐*value
intercept	2.288	0.256	< 0.001
$T_{1}$	0.133	0.193	0.492
$T_{2}$	−0.710	0.191	< 0.001
$T_{3}$	−1.437	0.327	< 0.001
gender	−0.298	0.216	0.167
stress	0.019	0.015	0.208
depression	0.045	0.040	0.254
presentation	−0.013	0.144	0.925
memory	0.277	0.180	0.125

**TABLE 6:** Coefficients from the model for the cry gestalt.

	Coefficient	SE	*p*‐value
intercept	−0.698	0.411	0.089
$T_{1}$	0.103	0.358	0.773
$T_{2}$	0.222	0.325	0.495
$T_{3}$	1.206	0.434	0.005
gender	0.017	0.258	0.947
stress	0.030	0.023	0.191
depression	0.072	0.050	0.152
presentation	1.120	0.245	< 0.001
memory	−1.152	0.334	< 0.001

Relative to codelength, the number of smile/laughter gestalts fell from the prenatal period toward the postnatal measurement points (each *p* < 0.001, Table 5). Conversely, the relative number of cry gestalts increased at the follow‐up measurement point (3y), compared to the prenatal period (*p* = 0.005). There is no notable trajectory of the relative number of movements within the prenatal period.

**FIGURE 4:**
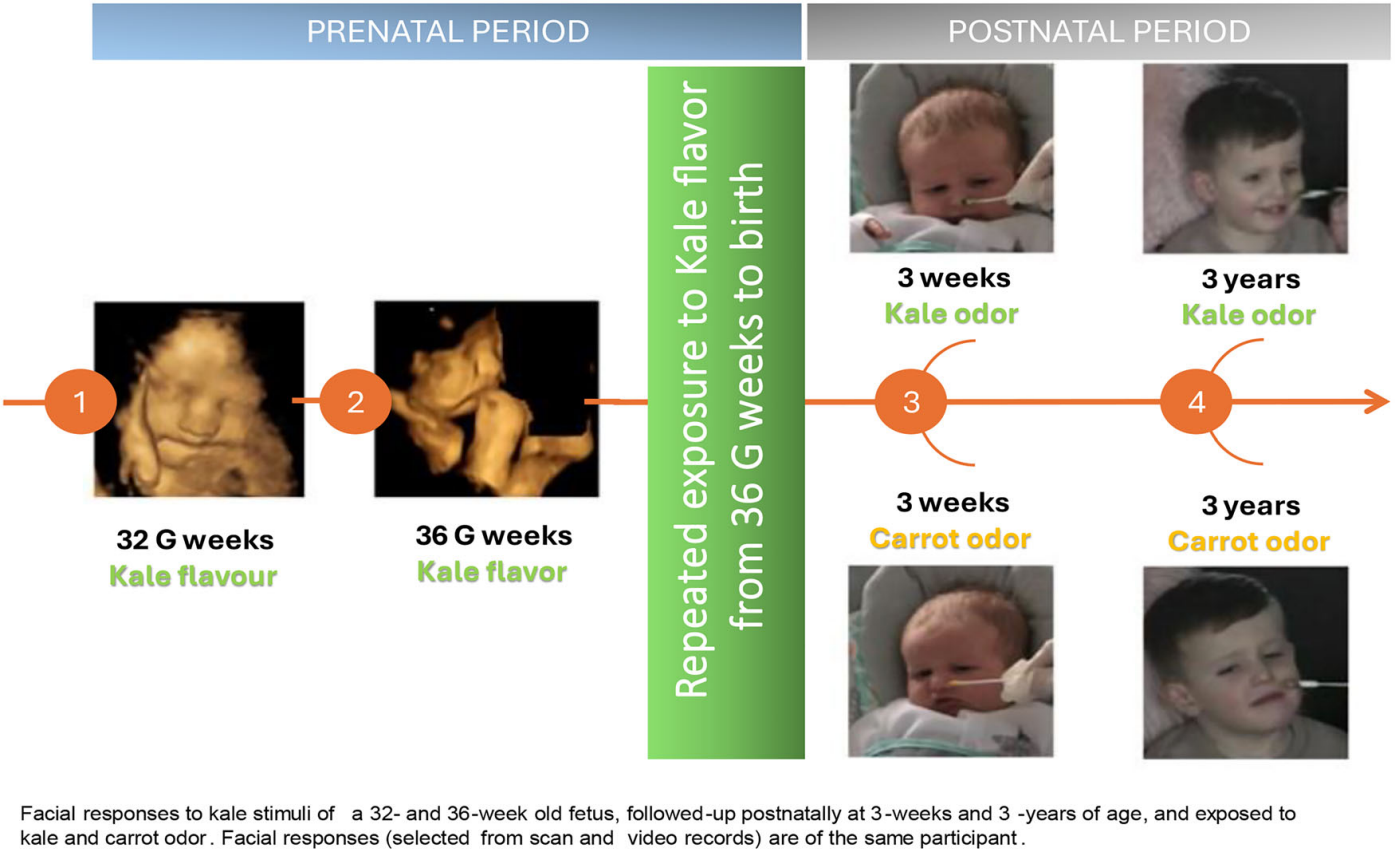
An example of facial responses to the kale stimulus of one fetus at 32‐ and 36‐week gestation, followed‐up postnatally at 3 weeks and 3 years of age, while being exposed to kale and carrot odors. The facial responses, selected from the ultrasound scans and video recordings, are of the same participant.

Across all measurement points, there is a general tendency for kale flavor to produce more cry‐face gestalts (*p* < 0.001; Table 6). However, if the flavor presented at postnatal measurement points matches the prenatal flavor stimulation and subsequent exposure (to either kale or carrot; see, e.g., Figure 4 of one child stimulated prenatally with kale), there is a reduction in the frequency of a cry‐face gestalt expression (*p* < 0.001; Table 6). This is in line with the previous observation that more cry gestalts appear to be expressed if the postnatal presentation is different from the prenatal experience (see Figure 2).

Neither maternal depression nor stress had a significant effect on the expression of gestalts in the postnatal period, even though the fact that both stress and depression consistently, albeit non‐significantly, increase the presence of both cry and smile/laughter gestalts may still be considered notable. There were no effects of fetus/infant gender on gestalt expression.

It is worth noting that, when omitting the memory variable from these models, the estimates of the remaining coefficients stay similar to the ones reported in Tables 5 and 6. However, while an analysis of deviance indicates no difference in goodness‐of‐fit between the respective models for the smile/laughter gestalts (*p* = 0.124), it does indicate a significant difference for the cry gestalts (*p* = 0.0008). These results, which can be extracted from the output file provided in the Supporting Information, render further credibility to the outcomes of the modeling. In summary, prenatal stimulation and subsequent exposure to flavors significantly affects postnatal reactions to the odor experienced prenatally, whereas maternal mental health does not show a significant effect in the present study.

## 4 Discussion

Regarding the long‐term effects of pre‐natal flavor exposure, the present research indicates that children, 3 years after exposure in the womb, respond less negatively to the odor component of the flavor they were repeatedly exposed to in the last trimester of pregnancy as compared to the flavor not ingested via calorie‐controlled capsules by their mothers. Thus, exposure to given flavors in utero shapes sufficiently stable flavor‐specific fetal memories, to be actualized in hedonic FM patterns elicitable by the sole odor corresponding to the flavor they were originally exposed to in utero. The finding that infant preferences are influenced by maternal dietary exposures from late prenatal and postnatal periods has been demonstrated in previous studies (e.g., Schaal 2023; Spahn et al. 2019; Ustun et al. 2023; Ventura et al. 2021). Extending this literature, our study shows that fetuses in the third trimester exhibit differential facial reactions to a bitter (kale) and a non‐bitter (carrot) flavor, and this differential reaction persists after birth; infant reactions at mean age of 3 weeks and later responses at 3 years of age reflected their prenatal reactions. Importantly, we observed a significant reduction in facial expressions, which could be interpreted as rejection of that vegetable odor when the child had been exposed to it repeatedly in the late stages of pregnancy.

Given previous research, which reported that maternal mental health affected fetal behavioral profiles (e.g., Reissland et al. 2020; 2021), in the current study, we investigated the effects of maternal stress, depression, and anxiety on fetal, neonatal, and infant smile/laughter and cry gestalt expressions when exposed to different flavors/odors. Although maternal mental health factors played a role, in that both stress and depression consistently but non‐significantly increased the presence of both cry and smile/laughter gestalts, it is not significant in the current sample. Offering healthy nutrition and hence exposure to healthy food flavors in infancy and childhood is essential for health in later life (e.g., Black et al. 2017; Schwartz et al. 2011). In contrast to our study, which examined fetal facial expressions in relation to stimulation with bitter and non‐bitter flavors, some research (e.g., Keresztes et al. 2022) argues that maternal mental health factors affect the development of healthy eating practices and hence exposure to healthy food types. Specifically, with increasing maternal mental stress, depression, and anxiety, the food environment in the home deteriorated including more use of counter‐indicated child feeding practices. However, summarizing results of studies on the effects of maternal mental health on feeding practices rather than exposure, Blissett et al. (2006) suggested that the relationship between maternal mental h*e*alth factors and child feeding practices is often ambiguous. Some studies report an effect of maternal anxiety but not depression (e.g., Farrow and Blissett 2005), others find an effect for both depression and anxiety (e.g., Coulthard and Harris 2003), and yet others find no maternal mental health effect at all on flavor preference (e.g., Whelan and Cooper 2000).

In sum, it is important to note that flavor preferences and dietary habits are influenced by a complex interplay of genetic, environmental, and cultural factors. For example, genetic differences among individuals play an important role in how various foods and odors are experienced. Therefore, genetic predispositions, causing various levels of sensitivity for flavors may change the importance of prenatal exposure effects. The reasons for not finding any significant interactions with maternal mental health factors and reactions to flavors could be manifold. For example, and still to be investigated is, whether the initial pregnancy exposure facilitated additional exposure to that vegetable across time. Some research suggests that weaning with vegetables, such as green beans, may promote vegetable acceptance in infants (e.g., Barends et al. 2013). In the present study, we cannot distinguish between fewer cry gestalts shown by 3‐year‐olds to the odor experienced prenatally as a flavor because they “remembered” prenatal exposure or whether fetal memory simply drove the “next step,” namely, better acceptance of the odor long term.

One strength of our study is that, in contrast to previous work where mothers were asked to consume flavors and postnatally infants of these mothers were tested on these odors or flavors, we observed fetal responses to either kale or carrot flavor and followed these fetuses to 3 years of age and then tested them on odors of the flavors experienced prenatally. However, a weakness of the study is that we were only able to follow a small number of participants from the fetal stage to 3 years of age. Also, testing neonates just after birth without the intervening 3 weeks when they might have had breastmilk exposure to either kale or carrot might have given us even stronger postnatal results. Additionally, we did not test for other factors, including genetic influences on taste perception, which might have influenced our results.

In conclusion, prenatal repeated exposure to a vegetable flavor reduces negative facial expressions in response to the odor of that vegetable 3 years later. This has the potential to influence the developmental trajectory of acceptance of a specific vegetable, given that caregivers often use an infant's or child's facial expression in response to foods as a primary cue to determine whether to offer a vegetable again (Forestell and Mennella 2012). Further work is needed to examine whether the effect of prenatal exposure on children's facial responses to vegetable odors has a meaningful effect on vegetable consumption.

## Funding

The longitudinal follow‐up was supported by an Aston University grant to J. Blissett and N. Reissland.

## Ethics Statement

This study was conducted in accordance with the Declaration of Helsinki, and ethical permission was granted by Durham University (PSYCH‐2019‐03‐12T15_59_32‐wvgf27). All participating mothers provided informed written consent for both themselves and their infants.

## Conflicts of Interest

The authors declare no conflicts of interest.

## Supporting information




**TABLE S1:** Fetal facial movements (FM) coded, and the configurations of facial gestalts.

## Data Availability

The SPSS data are available on request.
