# Supplementary material for: Do Human Fetuses Form Long‐Lasting Chemosensory Memories? Longitudinal Follow‐Up From Fetus to Young Child of Facial Responses to Flavor/Odor Stimuli
Source: Dev Psychobiol. 2026 May 12;68:e70165. doi: 10.1002/dev.70165 (PMC13167224; doi:10.1002/dev.70165)
Supplement: Supplementary file 1 — TABLE S1: Fetal facial movements (FM) coded, and the configurations of facial gestalts. [file DEV-68-e70165-s001.docx]

**Supplementary Table 1.** Fetal facial movements (FM) coded, and the configurations of facial gestalts.

| **Fetal facial movements** | **Laughter-face-gestalt** | **Cry-face-gestalt** |
| --- | --- | --- |
| FM1-Inner-brow-raiser |  | **✓** |
| FM2-Outer-brow-raiser* | **✓** | **✓** |
| FM4-Brow-lowerer |  | **✓** |
| FM6-Cheek-raiser | **✓** | **✓** |
| FM9–Nose-wrinkle | **✓** | **✓** |
| FM10–Upper-lip-raiser |  | **✓** |
| FM11–Nasolabial-furrow | **✓** | **✓** |
| FM12–Lip-corner-puller | **✓** |  |
| FM16–Lower-lip-depressor |  | **✓** |
| FM18–Lip-pucker | **✓** | **✓** |
| FM19–Tongue-show | **✓** |  |
| FM20–Lip-stretch |  | **✓** |
| FM24–Lip-presser | **✓** | **✓** |
| FM25–Lips-parting | **✓** | **✓** |
| FM26–Jaw-drop | **✓** | **✓** |
| FM27-Mouth-stretch | **✓** | **✓** |
| FM28-Lip-suck | **✓** | **✓** |
